# The Dynamic Impact of the COVID-19 Pandemic on Air Quality: The Beijing Lessons

**DOI:** 10.3390/ijerph18126478

**Published:** 2021-06-15

**Authors:** Chenlu Tao, Gang Diao, Baodong Cheng

**Affiliations:** School of Economics and Management, Beijing Forestry University, Beijing 100083, China; taochenlu@bjfu.edu.cn (C.T.); diaogang2021@outlook.com (G.D.)

**Keywords:** air quality, COVID-19, Beijing, TVP-VAR

## Abstract

Air pollution is one of the major environmental problems that endanger human health. The COVID-19 pandemic provided an excellent opportunity to investigate the possible methods to improve Beijing’s air quality meanwhile considering Beijing’s economic impact. We used the TVP-VAR model to analyze the dynamic relationship among the pandemic, economy and air quality based on the daily data from 1 January to 30 August 2020. The result shows that the COVID-19 pandemic indeed had a positive effect on air governance which was good for human health, while doing business as usual would gradually weaken this effect. It shows that the Chinese authority’s production restriction effectively deals with air pollution in a short period of time since the pandemic is just like a quasi-experiment that suddenly suspended all the companies. However, as the limitation stops, the improvement decreases. It is not sustainable. In addition, a partial quarantine also has a positive impact on air quality, which means a partial limitation was also helpful in improving air quality and also played an important role in protecting people’s health. Second, the control measures really hurt Beijing’s economy. However, the partial quarantine had fewer adverse effects on the economy than the lockdown. It is supposed to be a reference for air governance and pandemic control. Third, the more the lag periods were, the smaller their impact. Thus, restrictions on production can only be used in emergencies, such as some international meetings, while it is hard to improve the air quality and create a healthy and comfortable living environment only by limitation in the long-term.

## 1. Introduction

Air pollution is one of the major environmental problems that endanger human health [1,2,3,4,5]. Rapid economic development has promoted motor vehicles and industrial activities and resulted in serious air pollution [6]. PM_2.5_, with a particulate matter concentration of less than 2.5 μm, is one of the most important factors from the health perspective [7]. It is said that exposure to high PM_2.5_ caused more than 3.7 million premature deaths over the world in 2012 [1]. Concerned with the damaging health impacts of haze, the public has demanded a cleaner and more satisfying environment [8,9].

Poor air quality remains a severe issue for Chinese cities, especially Beijing, due to its capital functions [10,11]. In 2013, Beijing had 52% of days where the air quality was unhealthy, and the proportion of heavy pollution days reached 16.2% [12]. Since then, frequent air pollution occurrences in Beijing even became a cause for panic and regularly emerged as the main topic in social media [13,14]. Lots of research in various fields paid attention to air quality, seeking to solve the issue [15,16,17]. In line with data from China Environmental Monitoring Center, Beijing achieved a historic breakthrough in 2020. That is, the annual average concentration of PM_2.5_ was only 38 micrograms/cubic meter. If the control measures such as locking down cities substantially improved the air quality, the implied health benefits should be compared with the possible economic loss [18] to formulate the urban development strategy accordingly.

The COVID-19 pandemic might significantly influence air quality since the control measures decreased air pollutant emissions [19,20,21,22]. In late January, public transport networks, some enterprises, schools, and entertainment venues were suspended, and some areas even locked down [23]. The Chinese New Year national holiday was extended, and residents were encouraged to work from home. Thus, the emission these days was relatively low comparing with recent years [24,25,26]. Until 10 February, the government allowed some industries to get back to work, while the resumption process was complicated. Beijing’s Gross Domestic Product (GDP) decreased by 6.6% in the first quarter of 2020 [27]. On 12 June, the coronavirus was detected again in salmon in the Beijing Xinfadi Market, and then, the second outbreak occurred. The enterprises in the Beijing-Tianjin-Hebei region were affected again but less than the first outbreak owing to the partial quarantine. As of 16 July, Beijing has basically reached 100% of industrial enterprises above the designated size, and the resumption rate of catering above the designated size is 91.1% [28]. It is crucial to consider the pandemic’s impact on air quality to find better ways to improve air pollution and protect human health since it is just like a precious ‘natural experiment’.

The COVID-19 pandemic provided an excellent opportunity to investigate the possible methods to improve Beijing’s air quality meanwhile considering Beijing’s economic impact. To provide a sound basis for the effective control of urgent air pollution, it is of great importance to investigate the impact of the pandemic on air quality, also considering the economy. Since the policies and economy change rapidly during the pandemic, this study used the TVP-VAR model to analyze the dynamic relationship among the pandemic, economy, and air quality based on the daily data from 1 January to 30 August 2020. The results will shed light on the impact of the pandemic on air quality considering the economy and guide future control strategies and policymaking in Beijing’s air governance.

The rest of the paper presents the methodology and data (Section 2), the dynamic impacts of COVID-19 on air quality in Beijing (Section 3). Finally, we conclude.

## 2. Methodology and Data

### 2.1. Methodology

The TVP-VAR model is capable of seizing the time-varying feature in the economy and its development [29]. Social policies and economic environments change swiftly during the COVID-19 pandemic, and the relationship among the pandemic, the economy, and the air quality is obviously varying as time goes on. Traditional quantitative analysis methods such as VAR and SVAR are suitable for the study of maintaining a constant relationship between variables. However, during the COVID-19 pandemic, which has both obvious time-varying characteristics, traditional methods are likely to miss key time-varying information. while the time-varying parameter autoregressive model can capture the relationship and characteristics of variables in various backgrounds [30], overcoming the burden of randomly selected rolling-window-size [31,32,33,34]. Moreover, the TVP-VAR model does not have the same variance assumption [35,36], which is more in line with the actual situation. Therefore, the research results are more realistic.

The basic vector autoregressive (VAR) model is as follows:

(1) $$Ay_{t}=M_{1}y_{t-1}+\ldots +M_{s}y_{t-s}+\mu_{t},t=s+1,\ldots ,n.$$

This paper involves a total of m variables. Therefore, $y_{t}$ is a m × 1 vector, $A,\;M_{1},\;\ldots ,\;Ms$ is a m × m matrix of coefficients, and $\mu_{t}$ is a structural impact, which is also a m × 1 vector. The above formula can be rewritten as the following form:

(2) $$y_{t}=B_{1}y_{t-1}+\ldots +B_{s}y_{t-s}+A^{-1}\Sigma \varepsilon_{t},\varepsilon_{t}\sim N\left(0,I_{m}\right).$$

Among them, $B_{i}=A^{-1}M_{i},\;i=1,2,\ldots ,s$. And have $\Sigma =\left[\begin{array}{cccc} \sigma_{1} & 0 & \cdots & 0\\ 0 & \ddots & \ddots & 0\\ \vdots & \ddots & \ddots & 0\\ 0 & \cdots & 0 & \sigma_{m} \end{array}\right]$

It can be rewritten as:

(3) $$y_{t}=X_{t}\beta +A^{-1}\Sigma \varepsilon_{t},X_{t}=Im⊗(y_{t-1}^{\prime },\ldots ,y_{t-s}^{\prime }).$$

Among them, ⊗ means Kronecker product, which is an operation between two matrices.

Furthermore, taking into account the time changes of the parameters, we obtained the time-varying parameter vector autoregressive (TVP-VAR) model [36]. The model form is as follows:

(4) $$y_{t}=X_{t}\beta_{t}+A_{t}^{-1}\Sigma_{t}\varepsilon_{t},t=s+1,\ldots ,n$$

In the formula, the coefficient $\beta_{t}$, parameter $A_{t}$ and matrix $\Sigma_{t}$ all change with time. Let $\alpha_{t}$ denote the stacked vector of lower triangular elements in matrix $A_{t}$, and H be the logarithmic random volatility matrix. Suppose $h_{jt}=ln\sigma_{jt}^{2}$, and for all j=1,…,m, t=s+1,…,n, the parameters of the TVP-VAR model obey random walks. Suppose they are first-order random walk processes, which is

$$\left\{\begin{array}{c} \beta_{t+1}=\beta_{t}+\mu_{\beta t}\\ \alpha_{t+1}=\alpha_{t}+\mu_{\alpha t}\\ h_{t+1}=h_{t}+\mu_{ht} \end{array}\right..$$

Assume that $\varepsilon_{t}$, $\mu_{\beta t}$, $\mu_{\alpha t}$, $\mu_{ht}$ obey:

$$\left[\begin{array}{c} \varepsilon_{t}\\ \mu_{\beta t}\\ \mu_{\alpha t}\\ \mu_{ht} \end{array}\right]\sim N\left(0,V\right),\;\mathrm{thereinto},\;V=\left[\begin{array}{cccc} I_{k} & 0 & 0 & 0\\ 0 & \Sigma_{\beta } & 0 & 0\\ 0 & 0 & \Sigma_{\alpha } & 0\\ 0 & 0 & 0 & \Sigma_{h} \end{array}\right],$$

where $\beta_{s+t}\sim N(\mu_{\beta 0},\Sigma_{\beta 0})$, $\alpha_{s+t}\sim N(\mu_{\alpha 0},\Sigma_{\alpha 0})$, $h_{s+t}\sim N(\mu_{h0},\Sigma_{h0})$. Assume that the impacts of time-varying parameters are uncorrelated and that $\Sigma_{\beta 0}$, $\Sigma_{\alpha 0}$, $\Sigma_{h0}$ are all diagonal matrices. The estimation of the model in this paper is done by Markov Chain Monte Carlo (MCMC) method [35], which realizes dynamic simulation in which the sampling distribution changes as the simulation progresses.

### 2.2. Data

The phase of the epidemic can be judged based on changes in the number of confirmed COVID-19 cases. Therefore, this paper selected the number of newly confirmed COVID-19 cases on Beijing from January to August 2020 to represent the COVID-19 pandemic (new). The data were retrieved from the “Daily Epidemic Bulletin” of Beijing Municipal Health Commission (available online at http://www.nhc.gov.cn/xcs/yqtb/list_gzbd.shtml accessed on 25 September 2020).

This article used the daily passenger flow of Beijing Subway to represent Beijing’s economy (pas). According to the statistics of the “Beijing Transport Development Annual Report”, the passenger flow of Beijing rail transit was 3.85 billion passengers in 2018, accounting for 49.5% of the total urban passenger transport [37]. The subway is one of the main modes of transportation for residents in Beijing. Therefore, since most schools in Beijing closed from January to August 2020, the passenger flow of subway reflected the economic operation and the start of enterprises in Beijing. The data came from the daily passenger flow information published on the official Weibo website of Beijing Subway (available online at https://weibo.com/bjsubway accessed on 7 October 2020).

This article used PM_2.5_ to represent air quality in Beijing (pek). PM_2.5_ is one of the critical standards for measuring air quality, and the Beijing-Tianjin-Hebei region is an area with serious PM_2.5_. Therefore, this article extracted the hourly data of PM_2.5_ in Beijing and surrounding cities, calculated the daily data, and analyzed the relationship between the pandemic and air quality in Beijing. The data came from China Environmental Monitoring Center (available online at http://www.cnemc.cn/ accessed on 1 September 2020).

This paper used multiple imputation methods to impute and fill missing values in the data to ensure the validity and accuracy of the results. Since the data used in this article was daily data from 1 January 2020 to 29 August 2020, including the four seasons of spring, summer, autumn, and winter, the seasonality and periodicity of the data should be considered. Therefore, we adjusted the seasonally by subtracting the average data of the corresponding month in 2019 from the daily data in 2020 (see Table 1, the descriptive statistics of original data is shown in Appendix A).

## 3. The Dynamic Impacts of COVID-19 on Air Quality in Beijing

### 3.1. Unit Root Test

The sequences at level of three variables were stationarity. The stationarity of time series data was very important. We used the augmented Dickey–Fuller (ADF) test to check the stationarity of our series. the maximum lag order was 14 [38]. As mentioned in Table 2, the three series of the COVID-19, Beijing’s economy, and air quality were all stationary, which met the data requirements of the TVP-VAR model.

### 3.2. Estimation Results by MCMC

**Figure 1:**
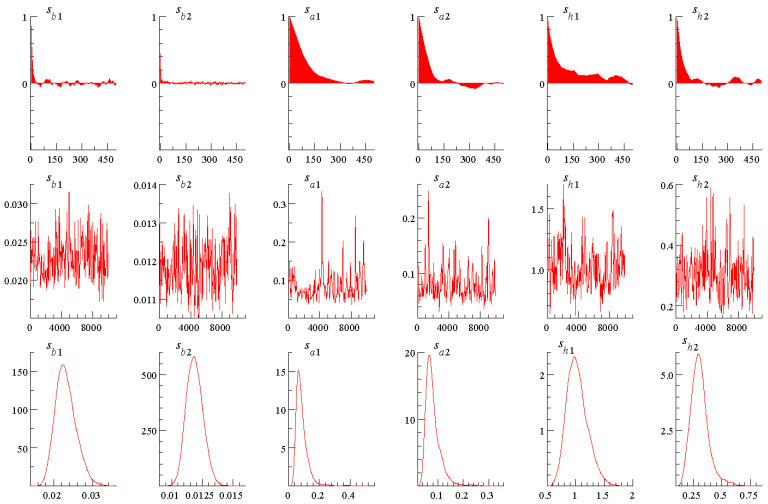
Estimation Results of the TVP Regression Model for the Simulated Data.

The SBIC and HQIC provide a consistent estimate of the correct lag order [39], so we used 1 as the lag order in this paper. We set the initial values of the parameters: $\mu_{\beta_{0}}=\mu_{\alpha_{0}}=\mu_{h_{0}}=0$, $\Sigma_{\beta_{0}}=\Sigma_{\alpha_{0}}=10I$, $\Sigma_{h_{0}}=100I$, ${\left(\Sigma_{\beta }\right)}_{t}^{-2}∼\gamma \left(40,0.02\right)$, ${\left(\Sigma_{\alpha }\right)}_{t}^{-2}∼\gamma \left(4,0.02\right)$, ${\left(\Sigma_{h}\right)}_{t}^{-2}∼\gamma \left(4,0.02\right)$, referred to Nakajima et al. [35]. Then we executed the MCMC algorithm for 10,000 samplings and discarded the first 1000 samplings by OxMetrics which is developed by Jurgen Doornik and David Hendry, then getting valid samples for model posterior estimation. It can be seen in the first line of Figure 1, in the displayed 500 samples, the autocorrelation of the sample decreased steadily. As for the second line, we found that each variable’s sample value fluctuates around the mean, which was not entirely random. The third line showed the density function of the posterior distribution. In general, Figure 1 showed that after the samples were discarded in the burn-in period, the degree of autocorrelation of the variables declined, meaning that it could usefully generate uncorrelated samples and ensure the exactitude of the results.

### 3.3. Time-Varying Impulse Analysis of Equal Interval

**Figure 2:**
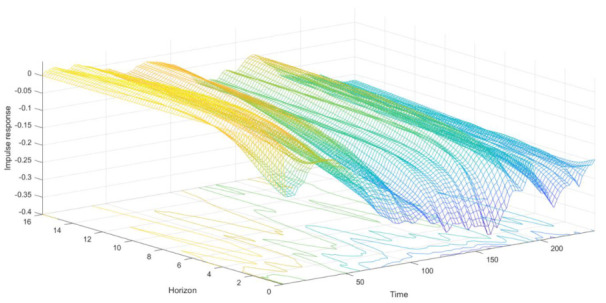
Time-varying responses of air quality to Beijing’s pandemic shock during the pandemic. Note: The Time-axis donates the date from January 2020 to August 2020, the Horizon-axis represents the impulse response horizons, and the Impulse-response-axis indicates the impulse response values after the shock of one-unit standard deviation.

**Figure 3:**
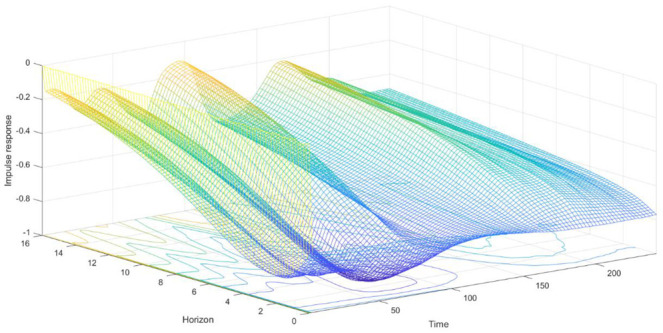
Time-varying responses of economy to Beijing’s pandemic shock during the pandemic. Note: The Time-axis donates the date from January 2020 to August 2020, the Horizon-axis represents the impulse response horizons, and the Impulse-response-axis indicates the impulse response values after the shock of one-unit standard deviation.

**Figure 4:**
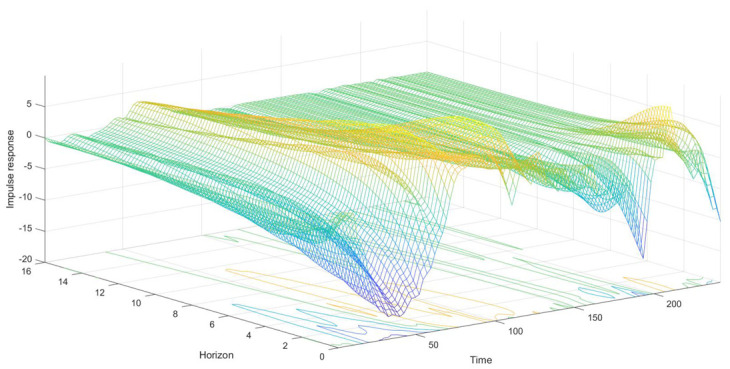
Time-varying responses of air quality to the economic shock during the pandemic. Note: The Time-axis donates the date from January 2020 to August 2020, the Horizon-axis represents the impulse response horizons, and the Impulse-response-axis indicates the impulse response values after the shock of one-unit standard deviation.

The parameter estimated of the TVP-VAR model changed over time. We could compare and analyze the difference of the influence of dependent variables at different time points by the impulse analysis of equal interval (also called impulse analysis of different time horizons). Figure 2, Figure 3 and Figure 4 showed the dynamic impulse response. We just took the impulse responses of equal intervals for a one-day horizon, a one-week horizon, and a two-week horizon, which represented the short-term, mid-term, and long-term, respectively, as an example. Moreover, according to Figure 2, Figure 3 and Figure 4, the impact of COVID-19 on air quality had obvious time-varying characteristics, which may be due to differences in control measures of the pandemic in different phases. Therefore, we used the impulse response of different time points to analyze the differences in the relationship among the COVID-19 pandemic, economy, and air quality in Beijing in different backgrounds. We took 22 January, 30 March, and 17 June 2020 as examples since they were in different stages of the COVID-19. January 22 was in the outbreak period of the pandemic. On 30 March, the number of newly confirmed cases of COVID-19 in mainland China went down to 3, which was much lower than the number of newly cured cases. However, on 17 June, the number of newly confirmed cases of COVID-19 in mainland China rose again.

Figure 2 shows that air quality’s impulse responses to the COVID-19 pandemic shock were below zero. The mid-term and long-term impulse response values were weaker than the short-term impulse response values, but the overall trends were consistent. In terms of all short-term effects, at the beginning of 2020, the pandemic had a small impact on air quality, with the short-term impulse responses at around −0.1, and the mid-term and long-term around 0. As time went by, the impulse responses gradually deepened, and the short-term impulse responses even reached about −0.25 in April. It was not until early April that the impact began to weaken. In mid-June, the impulse responses of Beijing air quality to COVID-19 shock deepened again. We knew that at the beginning of 2020, the pandemic had an impact on Beijing’s air quality, but the society had insufficient awareness of the pandemic and did not immediately adopt strict measures. Therefore, the COVID-19 pandemic led to an improvement in Beijing’s air quality, but the influence was small. At the end of January, the absolute values of impulse responses gradually increased. It means that most parts of China implemented the “lockdown” policy. Although Beijing announced that it would not lockdown, it also extended the Spring Festival holiday. The shutdown due to the pandemic also led to a reduction in industrial and exhaust emissions, which has further strengthened air quality optimization. At the beginning of April, it was at the point of partial to whole resumption. Emissions and pollution caused by getting back to production weakened the effect of the pandemic on air purification. In mid-June, due to the salmon incident in the Beijing Xinfadi Market, the pandemic repeated. The application of the “partial quarantine” policy once again strengthened the optimizing effect of the pandemic on Beijing’s air quality. According to the impulse response results, COVID-19 did have a positive effect on air governance, and getting back to business weakened this effect. It was consistent with the results of He et al. [18]. In addition, it is obvious that in January and February, the impact of the pandemic had a little impact on Beijing’s air quality, smaller than that in May and June. It shows that the anti-pandemic policy of partial quarantine was also of great help in improving air quality, and could provide a reference for air quality improvement.

The responses of air quality to the COVID-19 pandemic shock at three different time points were not wholly consistent but similar (Figure 2). The impulse response on 22 January converged faster than on 30 March and 17 June. It shows that on 22 January, the lagging impact of the pandemic on air quality was relatively small and short. It might be because Beijing did not implement strict pandemic control measures on 22 January. However, a similar trend indicates that the research results were robust.

Figure 3 shows that the impulse responses of the economy to the COVID-19 shock were also negative, and this effect decreased as the lag period increased. From the perspective of the short-term impulse responses, at the beginning of 2020, the impact of the pandemic on Beijing’s economy slightly increased. A turning point occurred in mid-March, and then, the impact gradually weakened. At the end of June, the impact of the pandemic on the economy deepened again. We found that initially, the impact of the pandemic on Beijing’s economy slightly deepened. It shows that the strict anti-pandemic policies implemented at the beginning of the year had an increasingly negative impact on population movements and economic operations. With the reopening plan, in mid-March, the negative impact of the pandemic on Beijing’s economy gradually diminished. In June, the pandemic in Beijing repeated, and the quarantine measures once again affected Beijing’s economy. However, compared with January, it was evident that the partial isolation policy in June had a less negative economic impact than the total shutdown of production in January and February. As for the medium and long term, the overall trends were the same as the short-term one, with a small influence but large fluctuations. In mid-term and long-term responses, the time when the impact of the repeated pandemic on the economy deepened became advanced. In mid-June, the absolute values of the impulse responses of the economy to pandemic shock increased, which also shows that the partial quarantine in June had a less negative economic impact than the policy in February. According to the impulse response results, the pandemic harms Beijing’s economy, which was consistent with He et al. [18]. Both the partial quarantine and the lockdown could control the pandemic, and the partial quarantine had fewer negative effects on the economy than the lockdown.

The impulse responses of the economy to COVID-19 shock at three different time points were generally consistent (Figure 3), which were all negative and became weak, while the response on 17 June was a little slower than the others. Because the second pandemic policy was partial segregation, which was looser than in the first pandemic, it shows that the partial quarantine policy had fewer negative effects on the economy than the total lockdown.

Figure 4 shows that the impulse responses of air quality to Beijing’s economic shock had obvious time-varying characteristics in the short term. The more the lag periods were, the smaller their impact. Almost all impulse responses converged near 0 after the seven-day horizon. Therefore, the relationship between the air quality and the economy in Beijing was mainly short-term. Initially, the short-term impulse responses of Beijing’s air quality to Beijing’s economic shock quickly dropped to around −13. In mid-March, the impulse responses increased over zero. In April and May, the impulse responses of Beijing’s air quality to Beijing’s economy stabilized at around 7.5. In June, the impulse responses again went down below zero and returned above zero in July. We knew that at the beginning of 2020, the impulse responses were negative. It shows that from January to early March, the shutdown and the initial economic reopening of Beijing were conducive to optimizing air quality. At that time, Beijing was implementing strict anti-pandemic policies, companies stopped work and production, and the traffic volume was also significantly reduced. Although some production began to resume, nature repaired itself [40,41]. From mid-March to May, the impulse responses of Beijing’s air quality to the economy increased. It shows that at that time, Beijing’s economic recovery aggravated the level of PM_2.5_, and it means that the environmental impact of getting back to production exceeded the tolerance of nature. In June, the impulse responses appeared negative again, but the absolute values of the impulse response were smaller than the first pandemic. It shows that the partial quarantine policy implemented in June was also conducive to reducing PM_2.5_, but the effect was weaker than the lockdown. According to the results of the impulse responses, the shutdown of work was indeed beneficial to the improvement of PM_2.5_, but production within the natural carrying capacity would not aggravate the deterioration of the environment.

We also took shocks at three different time points as an example. The changing trends of the impulse responses of the air quality to economic shock on 22 January and 17 June were basically similar, both below zero, and gradually became weaker. While the response on 30 March was above zero and then decreased. It was consistent with the findings of impulse analysis of equal intervals. From January to early March and June, Beijing’s economic recovery was conducive to the optimization of air quality. From mid-March to April, and July and August, Beijing’s economic recovery increased air pollution. Besides, the air quality on March 30 was less affected by the economic shock. It illustrates that the partial quarantine was also conducive to the reduction of PM_2.5_, but the effect was weaker than the lockdown.

## 4. Discussion

The COVID-19 pandemic led to an improvement in air quality and therefore benefited people’s health, consistent with the conclusions of Berman and Ebisu [42], Singh and Chauhan [43], Stratoulias and Nuthammachot [44], Nadzir et al. [45], Nakada and Urban [46], Ma and Kang [47], and Donzelli et al. [48]. They all presented evidence of the bettering of the air quality over large metropolitan areas during the COVID-19 pandemic. We also examined the dynamic impact of the pandemic on the economy, which had also been negative, but it was clear that the absolute value of the impulse response in February was larger than that in June. It explains that the negative economic impact of the second outbreak was not as great as the first one. It was related to the severity of the pandemic and the rationality of response policies. The influence of Beijing’s economy on PM_2.5_ was unstable, but in both outbreaks, the impulse responses were below zero since the economy improved air quality due to the control measures. In addition, these measures caused the shutdown of factories and traffic, and ultimately reduced pollution emissions. During the corresponding recovery periods, the impulse responses increased above zero. When the economy reopened, factories and road traffic resumed, and PM_2.5_ went up again. Therefore, Beijing’s air control measures should not be “one-size-fits-all” across the whole city but should be implemented separately by region. When the smog is severe, the authorities should limit the emissions by regions and stages, and it is supposed to be able to achieve similar significant results.

The application of the “partial quarantine” also strengthened the optimizing effect of the pandemic on Beijing’s air quality, the same as the lockdown. However, the partial quarantine in June had a less negative economic impact than the lockdown, consistent with He et al. [18]. Therefore, the anti-pandemic policy of partial quarantine was also of great help in improving air quality and could provide a reference for air quality improvement and health protection.

The mid-term and long-term impulse response values were weaker than the short-term impulse response values, but the overall trends of the three were consistent. Therefore, the research results were robust, and the more the lag periods were, the smaller the impact would be. Almost all impulse responses converged after the seven-day horizon. It illustrates that the relationship of the pandemic, the economy, and the air quality in Beijing was mainly short-term. Therefore, restrictions on production and emission can only be used as immediate policies to respond to emergencies, and it is difficult to produce long-term improvements to the environment and living conditions.

## 5. Conclusions

In order to find a possible way to improve Beijing’s air quality meanwhile considering the economic impact in Beijing, this article used the time-varying parameter vector autoregressive model based on the daily data of the pandemic and air quality from January to August 2020 to analyze the differences in the relationship between the pandemic, the economy, and air quality in Beijing during different phases of the pandemic. The research results show the following: First, the COVID-19 pandemic indeed had a positive effect on air governance which is good for people’s health, while doing business as usual would gradually weaken this effect. It may result due to the reduction of emissions from both production and traffic during the pandemic. It also shows that the Chinese authority’s production restrictions effectively deal with air pollution in a short period of time since the pandemic is just like a quasi-experiment that suddenly suspended all the companies. Moreover, local emission restrictions had a positive impact on air quality too. Thus, a partial limitation was also helpful in improving air quality and also played a role in protecting people’s health. Second, the pandemic hurt Beijing’s economy. Both the partial quarantine and the lockdown would control the pandemic, while the partial quarantine had fewer adverse effects on the economy than the lockdown. It is supposed to be a reference for air governance and pandemic control. Third, the more the lag periods were, the smaller the impact would be. It may be because the relationship between the pandemic and the air quality in Beijing was mainly short-term. Thus, restrictions on production can only be used in emergencies, and it is hard to improve the air quality for people’s health in the long term.

Based on the above conclusions and the status quo of Beijing, we propose some implications for Beijing’s air governance and pandemic prevention: As the pandemic has become the “new normal”, it is vital to find a good response that less influences the economy. The partial quarantine policy can effectively prevent and control the COVID-19 pandemic. At the same time, it can improve air quality with few negative effects on the economy. Therefore, improving the partial quarantine policy will help ensure the economy’s stable operation while controlling the pandemic. Moreover, Beijing’s air control measures should not be “one size fits all” across the whole city but should be implemented separately by region. When the smog is severe, the authorities should limit the emissions by regions and stages, and it is supposed to achieve similar significant results. Besides, we should also find more effective measures to improve air quality since emission restriction can only be used in emergencies.

## Figures and Tables

**Table 1:** Data description.

Variables	N	Mean	Min	Max	Std. Dev.
new	242	3.967	0	36	7.603
pas	242	−481.235	−850	170	231.252
pek	242	−2.018	−49	153	33.308

**Table 2:** Augmented Dickey–Fuller test.

	Variables	Without Constant and Drift	With Drift	With Constant and Drift
At Level	new	−3.046 ***	−3.915 ***	−4.301 ***
	pas	−0.526 ***	−3.069 ***	−4.232 ***
	pek	−2.870 ***	−2.837 ***	−2.837 ***

Note: *** *p* < 0.01, ** *p* < 0.05, * *p* < 0.10.

## Data Availability

All data used during the study are available from the corresponding author by request.

## Appendix A

**Table A1:** Data description.

Variables	N	Mean	Min	Max	Std. Dev.
new	242	3.967	0	36	7.603
pas	242	396.741	412	10265	248.790
pek	242	40.774	4	206	33.919
